# Traditional Applications, Phytochemistry, and Pharmacological Activities of *Eupatorium lindleyanum* DC.: A Comprehensive Review

**DOI:** 10.3389/fphar.2020.577124

**Published:** 2020-12-08

**Authors:** Xueyi Wang, Shangying Ma, Feifan Lai, Yiqi Wang, Chenghua Lou

**Affiliations:** Department of Pharmacology, College of Pharmaceutical Sciences, Zhejiang Chinese Medical University, Hangzhou, China

**Keywords:** Eupatorium lindleyanum DC, botany, pharmacology, toxicity, pharmacokinetics, phytochemistry

## Abstract

*Eupatorium lindleyanum* DC. (EL) has a long history of traditional use in China to cure coughs, chronic bronchitis, lobar pneumonia, and hypertension. Because of this extensive use of EL in traditional medicine, this present review gives a systematic overview of the conventional applications, phytochemistry, and pharmacological effects of the herb. Literature was systematically searched using the scientific databases ScienceDirect, SciFinder, CNKI, Wiley, Baidu Scholar, SpringerLink, PubMed, Web of Science, and other professional websites. Information was also gathered from books on traditional Chinese herbal medicine, the Chinese Pharmacopoeia and Chinese Materia Medica. To date, many preparations of EL have been widely used clinically to treat various diseases of the respiratory system. More than 100 compounds have been isolated from the herb, including triterpenes, sesquiterpenes, sesquiterpene lactones, flavonoids, acyclic diterpenoids, sterols, and so on. Among them, terpenoids are considered to be the most important bioactive substances in EL. The pharmacological functions of EL, including anti-asthmatic, anti-tussive, anti-inflammatory, anti-hyperlipidemic, anti-hypertensive, anti-virus, and anti-tumor activities, have been widely investigated. However, most of the studies are preclinical research. Further studies are required to examine the underlying mechanisms of action. Traditionally, EL is used for treating many diseases, especially respiratory diseases. Unfortunately, up to now, modern studies have not yet well elucidated the conventional usage of EL. Most importantly, its biological activities and the corresponding constituents are still unclear. Moreover, studies on the pharmacokinetics and toxicity of EL are few, so data on the clinical safety of EL are lacking. Taken together, research work on EL is quite preliminary. More in-depth studies of phytochemistry, pharmacological activities, pharmacokinetics, and toxicity of the herb are needed. This review aims to provide valuable information on EL to guide future investigations and applications.

## Introduction

**FIGURE 1:**
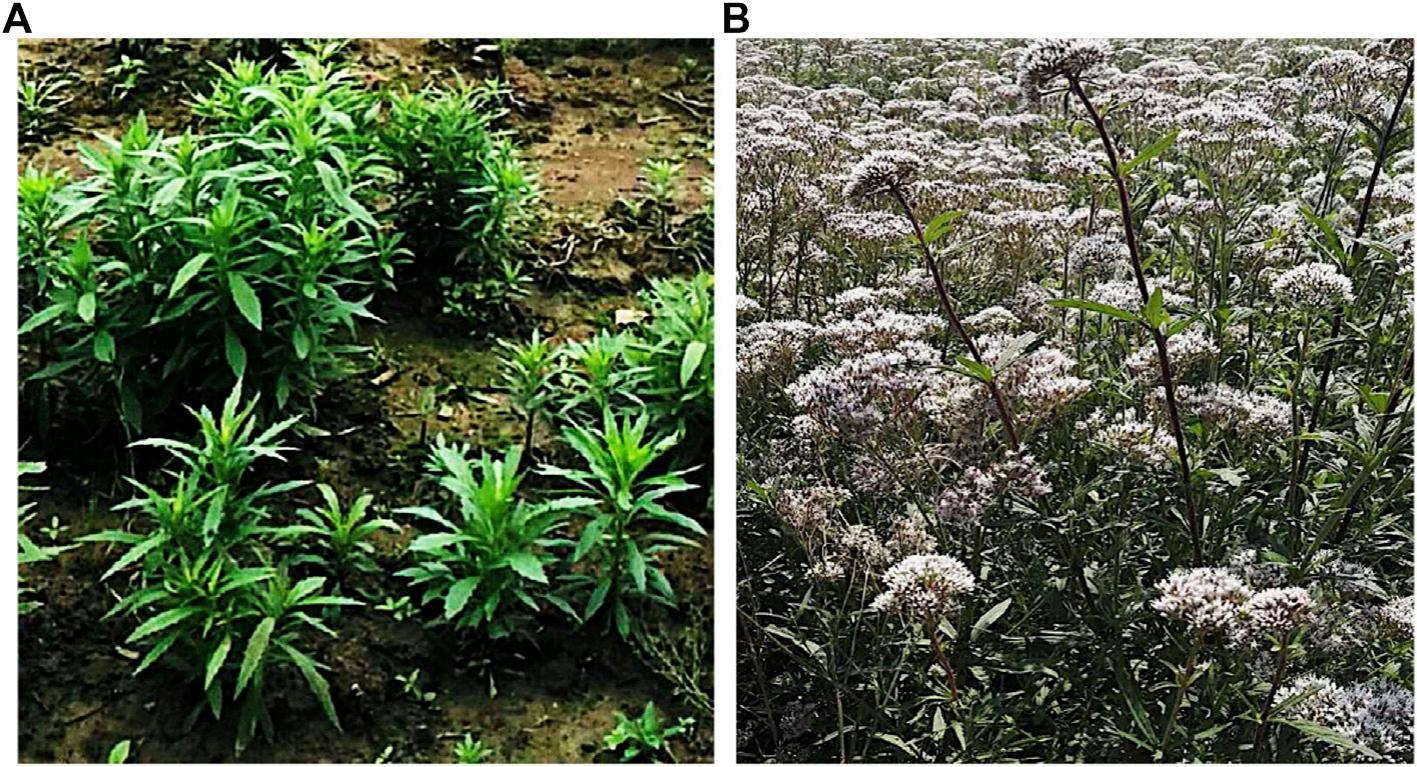
*Eupatorium lindleyanum* DC. **(A)** Seedlings **(B)** Plant.

*Eupatorium lindleyanum* DC. (EL) (Figure 1), also known as “Yemazhui,” has been traditionally used to treat coughs, chronic bronchitis, and hypertension for thousands of years in China (Editorial Committee of the Administration Bureau of Traditional Chinese Medicine, 1998). The plant is mainly distributed in the Chinese provinces of Jiangsu, Gansu, Shandong, and Hunan. Importantly, Jiangsu Province plant is considered to be an authentic herb. EL has been recorded in the China (Pharmacopoeia, 1977), the Jiangsu Provincial Standard of Local Medicinal Materials 1988, and the China National pharmacopoeia commission, 2015). It possesses the functions of reducing phlegm and relieving cough and asthma (National Pharmacopoeia Commission, 2015).

Over the last few decades, researchers have shown interest in the bioactive constituents of EL due to the extensive biological activities of this herb. The components of the plant have been widely investigated and more than 100 compounds have been isolated from the plant. Several types of constituents have been isolated from EL, such as sesquiterpenes, diterpenoids, triterpenoids, volatile oil, flavonoids, and so on (Ito et al., 1979; Yang et al., 2003; Huo et al., 2004; Qian et al., 2004; Yang et al., 2005a; Huo et al., 2006; Ye et al., 2008; Wu et al., 2012a). Also, numerous studies have demonstrated a great number of pharmacological functions of the constituents and fractions from EL, including anti-hyperlipidemic (Zhou et al., 2016), antioxidant (Yan et al., 2011), anti-cancer (Yang et al., 2007), anti-viral (Peng et al., 2008a), and anti-inflammatory effects (Chu et al., 2016). However, review articles about its phytochemistry and pharmacological characteristics are few. Therefore, we have undertaken to make a detailed review of EL by searching a variety of literature from different databases.

The present review was compiled from reported studies on the botany, traditional applications, identified compounds, pharmacological activities, toxicology, and pharmacokinetics of the herb. We also discuss the limitations of the current studies of EL and suggest areas of interest for potential future research. We expect that this review will be useful in providing valuable information for future in-depth investigations and applications.

## Botany

EL is a perennial herb that grows to a height of approximately 1–2 m. The rhizome is short with fibrous roots, and the stem is erect. The upper part is branched, light brown or purple, and scattered with purple spots. Young plants are covered in dense hairs. Leaves are usually opposite, 3-lobed verticillate, sometimes undivided or deeply divided, and sessile. Lobes are linear lanceolate, margins sparsely serrate, and hairy on both sides with glandular dots below. Leaves have three veins. The capitula are numerous with short stalks, the inflorescence is corymbose; the involucre is bell-shaped with nine lanceolate bracts and each capitulum has five tubular flowers, five-lobed apex, five stamens, and two-lobed stigmas. The tubular flowers are bisexual and purplish. Achenes are black, elliptic, and slightly oblate. Flowering occurs from July to September and fruiting from August to October (Editorial Committee of the Administration Bureau of Traditional Chinese Medicine, 1998; National Pharmacopoeia Commission, 2015).

## Traditional Applications

Because of the effective pharmacological functions of EL, there is a long history of usage of the plant in China. Anecdotally, the history of the herb for treating diseases can be traced back to the “Warring States Period” (475–221 BCE). At that time, it was mainly used to treat horses with respiratory diseases. It has been considered a “folk herb” since ancient times until it was recorded in the China Pharmacopoeia in 1977. According to the China Pharmacopoeia, EL possesses the function of reducing phlegm, clearing away lung heat, and relieving cough and asthma (Editorial Committee of the Administration Bureau of Traditional Chinese Medicine, 1998; National Pharmacopoeia Commission, 2015). Traditionally, it was usually used to treat lung heat, cough, chronic bronchitis, and lobar pneumonia.

The preparations of EL, such as Eupatorium Kechuan powder (野马追克喘散), modified Yemazhui capsule (加味野马追胶囊), sustained-release preparation of Yemazhui (野马追缓释制剂), and Yemazhui syrup (野马追糖浆), are widely used clinically to treat diseases of the respiratory system (Zhu et al., 2012; Su et al., 2016; Zhang, 2017). The applications of EL preparations are summarized in Table 1. In the early 1970s, EL was used to treat patients with chronic bronchitis (People’s Hospital of Xuyi in Jiangsu Province, 1973; Jiangsu Hospital of Jiangsu Province, 1976a). The flavonoids and alkaloids extracted from EL showed significant anti-asthmatic and anti-tussive activities, and markedly improved chronic bronchitis. Su et al. (2016) evaluated the effects of modified Yemazhui capsule in treating patients with acute bronchitis. Sixty patients were involved in the study. Results demonstrated that the modified Yemazhui capsule was effective in treating acute bronchitis. The clinical response rate (CRR) was 96.15%. Also, the symptom scores of the modified Yemazhui capsule-treated group were significantly improved (Su et al., 2016). Consistent with this, a study by Yan (2013) demonstrated that the symptoms of chronic bronchitis were markedly alleviated by treatment with Jiawei Yemazhui capsule. The CRR of Jiawei Yemazhui capsule reached 94.44% (Yan, 2013). Yemazhui syrup is another preparation of EL in common clinical use. Clinical investigations on the effects of Yemazhui syrup on children with chronic bronchitis were evaluated by Zhang (2017). Results demonstrated that the Yemazhui syrup was effective in alleviating chronic bronchitis (96.5%). Importantly, the preparation did not show any significant adverse reactions (Zhang, 2017). It was also reported that Yemazhui syrup was effective in treating patients with lobar pneumonia. Treatment with Yemazhui syrup significantly alleviated lung inflammation and normalized the body temperature and leukocyte counts.

**TABLE 1:** Clinical trials of EL related preparations.

Preparations	Patients	Treatment	Primary endpoint	Findings	Refs.
Modified Yemazhui capsule	52 patients aged 18–77 with acute or chronic bronchitis	Group 1 (n = 26): Modified Yemazhui capsule (0.6 g/time, t.i.d.) Group 2 (n = 26): Keke capsule (0.6 g/time, t.i.d.) Both groups were treated for one week	Disease symptoms The symptom scores	Modified Yemazhui capsule effectively improved clinical symptoms of bronchitis with a cure rate of 96.15%	Su et al. (2016)
Jiawei Yemazhui capsule	72 patients with chronic bronchitis	Group 1 (n = 36): Jiawei Yemazhui capsule (b.i.d.) Group 2 (n = 36): Keke capsule (0.6 g/time, t.i.d.) Both groups were treated for one week	Disease symptoms The symptom scores	Jiawei Yemazhui capsule was effective in treating patients with chronic bronchitis with the syndrome type of phlegm-heat obstructing lung with a cure rate of 94.44%	Yan (2013)
Sustained-release preparation of Yemazhui	200 roosters with bronchitis	Group 1–3 (n = 150, 50 roosters/group): Sustained-release preparation of Yemazhui (0.5, 1.0, and 1.5 g/time, b.i.d.) for 6 days; group 4 (n = 50): Manhu powder (0.5 g/time, b.i.d.) for 6 days	Disease symptoms Body weight	Sustained-release preparation of Yemazhui was effective in treating roosters with bronchitis with a cure rate of 82%	Wang et al. (2014)
Yemazhui syrup	400 children aged 2–12 with chronic bronchitis	Group 1 (n = 200): Yemazhui syrup (5–10 ml/time, b.i.d.) for one week Group 2 (n = 200): Ambrocol oral solution (8–15 ml/time, b.i.d.) All groups were treated for 10 days	Disease symptoms	Yemazhui syrup was effective in treating children with chronic bronchitis. The cure rate was 96.50%	Zhang (2017)
Yemazhui syrup	17 patients with lobar pneumonia	Yemazhui syrup (30 ml/time, t.i.d.) for one week	Disease symptoms Body temperature Lung inflammation Leukocyte counts	Yemazhui syrup was effective in treating patients with lobar pneumonia	Jiangsu Hospital of Jiangsu Province (1976b)
Yemazhui injection	32 patients aged 8–58 with leptospirosis	Yemazhui injection (4 ml/time, t.i.d.) for 3 days	Disease symptoms Body temperature	Yemazhui injection was effective in treating patients with leptospirosis. The cure rate was 96.80%	Yan (1987)
Flavonoid tablet Alkaloid tablet Flavonoid + alkaloid tablet	150 aged patients with chronic bronchitis	Flavonoid group (n = 20) Alkaloid group (n = 22) Flavonoid + alkaloid group (n = 108) All groups were treated for 20 days	Disease symptoms Pulmonary emphysema	Flavonoid + alkaloid tablets showed significant therapeutic effects in chronic bronchitis. The cure rate was 86.10%	People’s Hospital of Xuyi in Jiangsu Province (1973)

Abbreviations: EL, Eupatorium lindleyanum DC.; t.i.d., Three times a day; B.i.d., Two times a day.

Results from animal studies also support the above findings. Numerous experiments have documented the therapeutic effects of EL in treating animals with respiratory diseases. It was reported that the sustained-release preparation of Yemazhui showed potential effects in treating roosters with bronchitis (82%) (Wang et al., 2014). Eupatorium Kechuan powder was also demonstrated to be effective in treating diseases of the respiratory system in pigs (93.3%) (Zhu et al., 2012). The effects of EL injection in treating human patients with leptospirosis were reported by (Yan, 1987). According to the report, after treatment for 12.9 h, the body temperature of patients was normalized and the clinical cure rate was 96.8% (Yan, 1987).

The above clinical studies demonstrate the effectiveness of preparations of EL in treating diseases of the respiratory system, especially for bronchitis and lobar pneumonia. However, although the effects of EL in treating leptospirosis were reported, more clinical studies are still required to further verify this pharmacological activity.

## Phytochemistry

**FIGURE 2:**
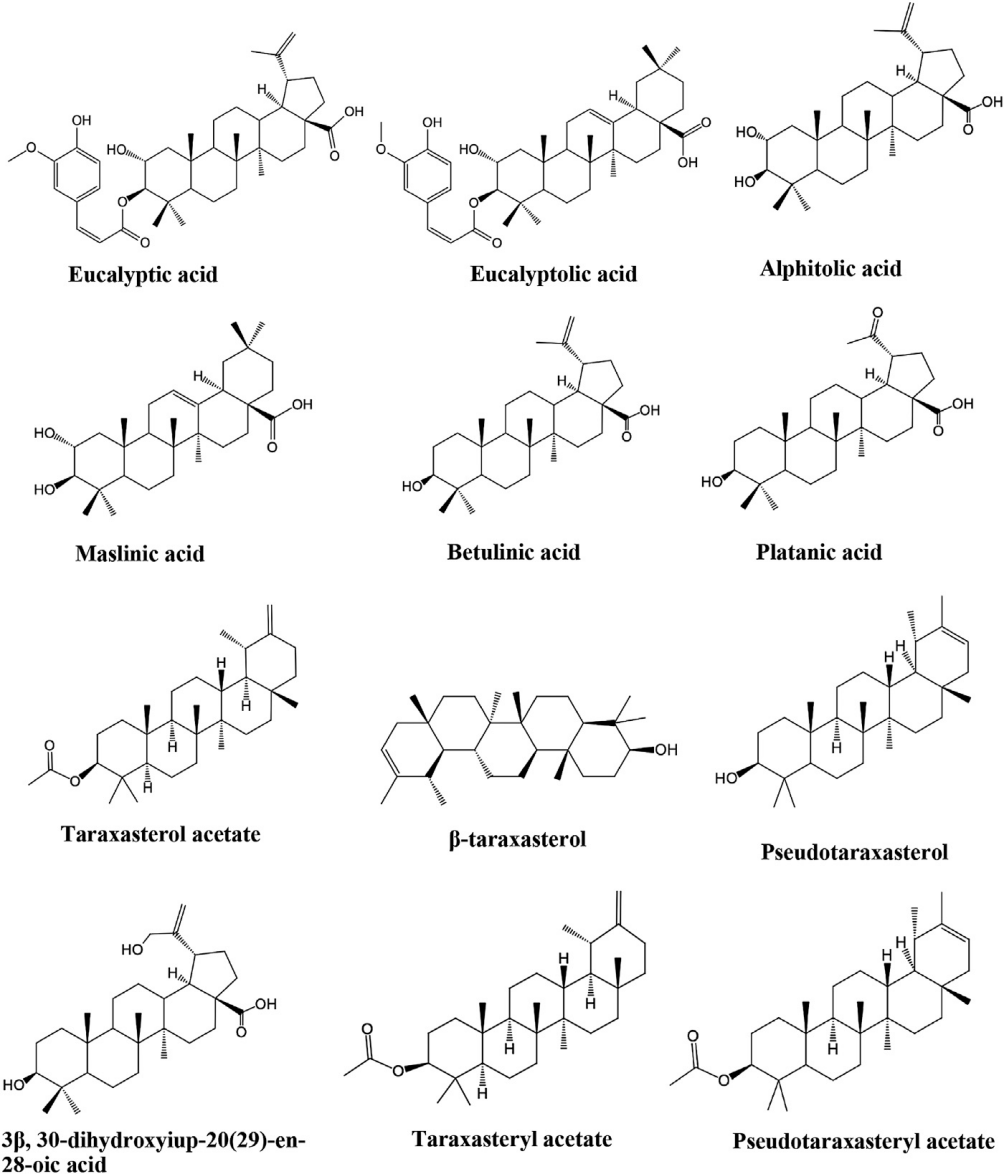
Identified triterpenes in EL.

**FIGURE 3:**
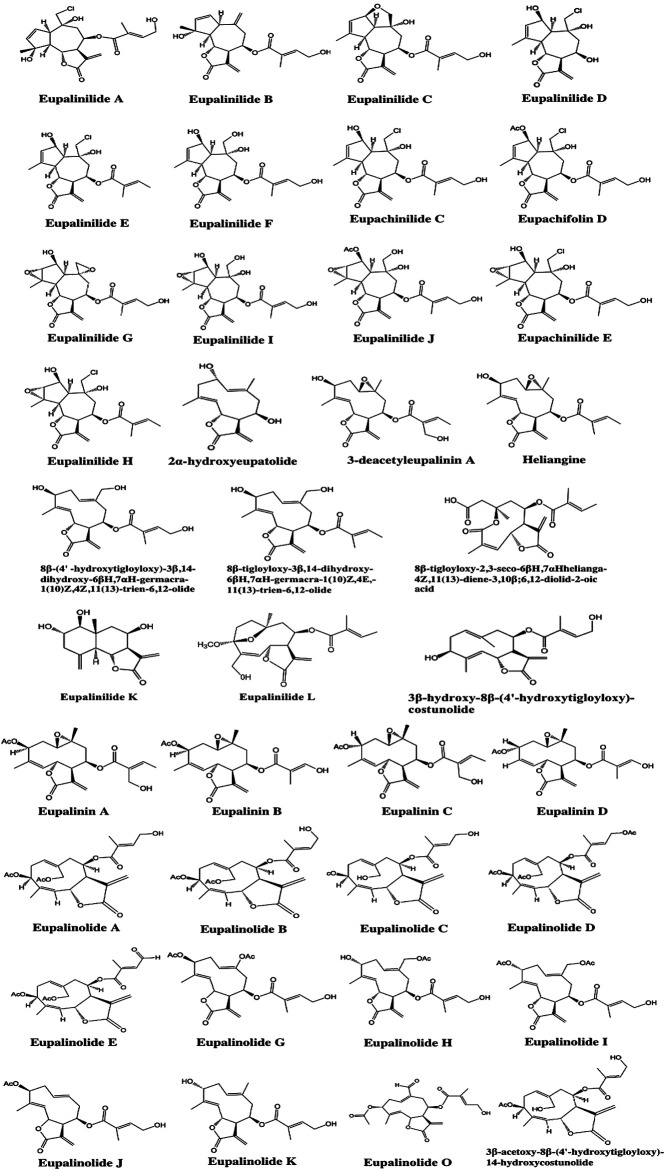
Identified sesquiterpenoids in EL.

**FIGURE 4:**
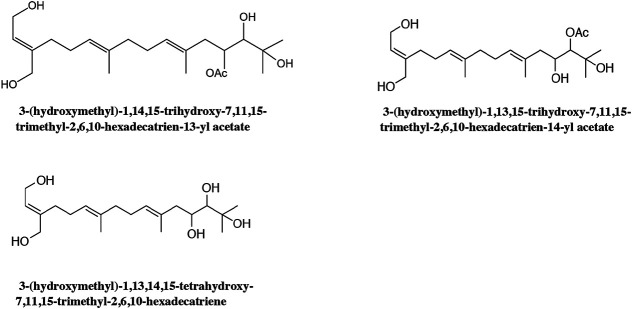
Identified acyclic diterpenoids in EL.

**FIGURE 5:**
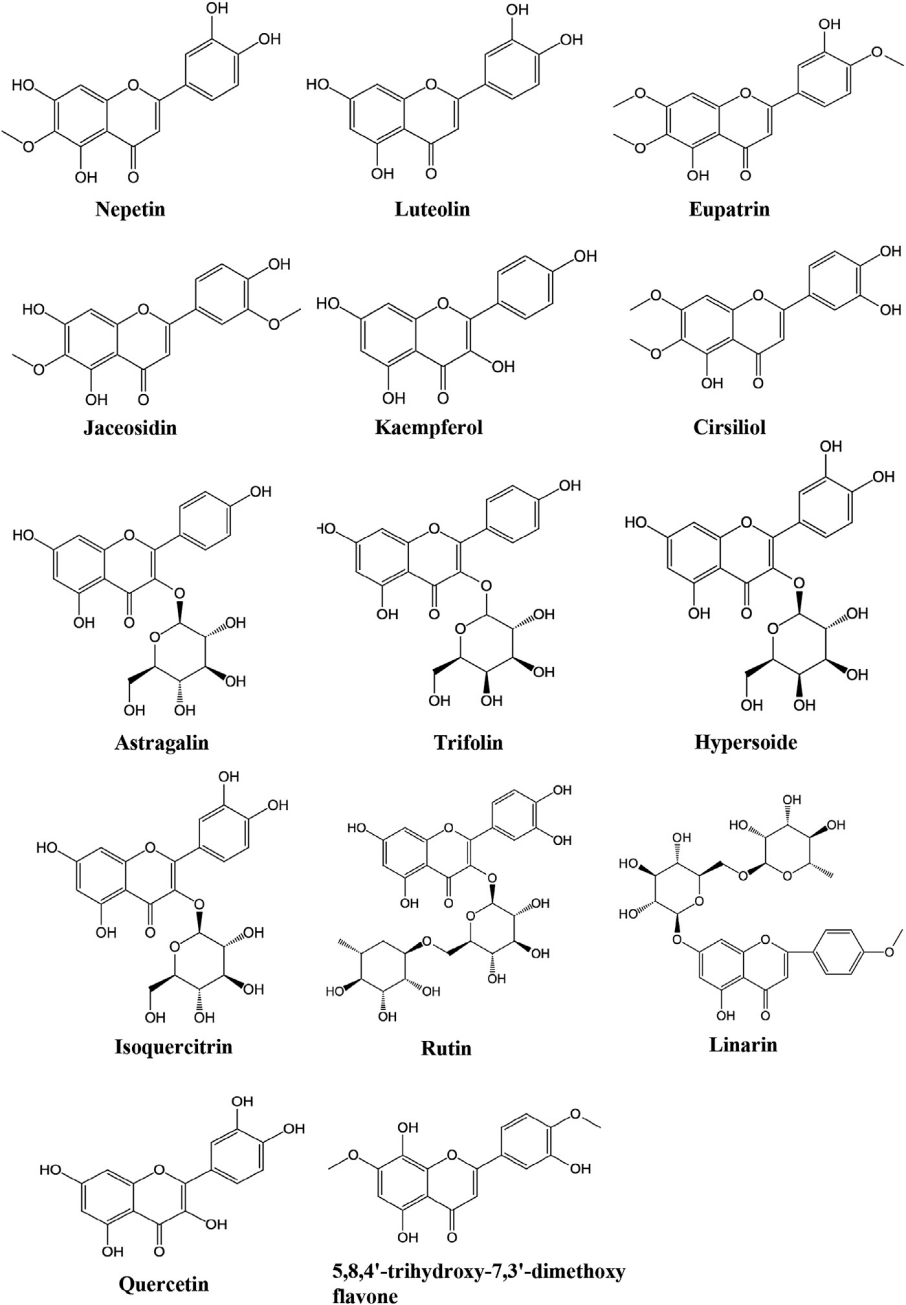
Identified flavonoids in EL.

**FIGURE 6:**
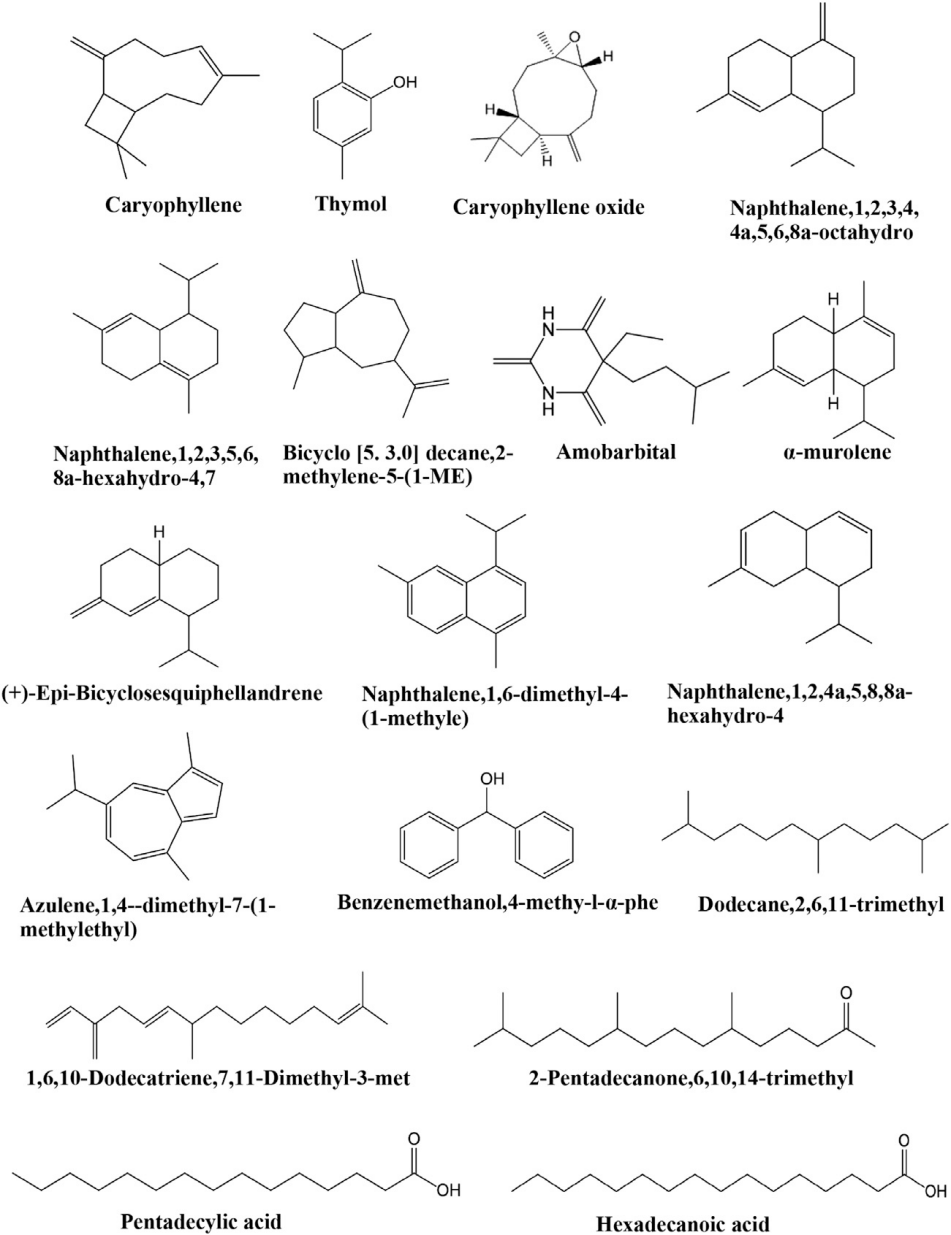
Identified compounds in volatile oil of EL.

**FIGURE 7:**
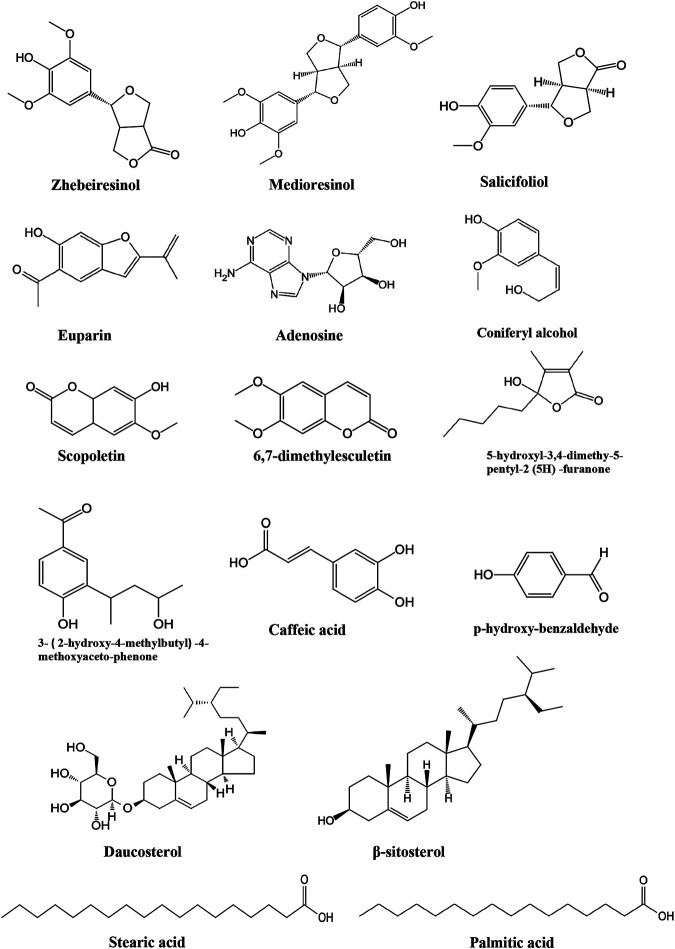
Other compounds identified in EL.

Due to the extensive use of EL in traditional Chinese medicine, the bioactive constituents and pharmacological functions of EL have been widely studied. Several categories of phytochemicals have been identified. To date, more than 100 components have been verified in EL, such as triterpenes, sesquiterpenes, sesquiterpene lactones, flavonoids, acyclic diterpenoids, sterols, alkaloids, and so on. Among them, terpenes are considered to be one of the most important constituents in this plant. Table 2 shows all the compounds isolated from EL. The chemical structures are illustrated in Figures 2–7.

**TABLE 2:** Chemical components of EL.

NO.	Chemical components	Formula	Plant part	Extraction methods	Refs.
Triterpenes					
1	Eucalyptic acid	C_40_H_56_O_7_	Whole plant	Ethanol extract (95% v/v)	Wang et al. (2013)
2	Eucalyptolic acid	C_40_H_56_O_7_	Whole plant	Ethanol extract (95% v/v)	Wang et al. (2013)
3	Alphitolic acid	C_30_H_48_O_4_	Whole plant	Ethanol extract (95% v/v)	Wang et al. (2013)
4	Maslinic acid	C_30_H_46_O_4_	Whole plant	Ethanol extract (95% v/v)	Wang et al. (2013)
5	Betulinic acid	C_30_H_48_O_3_	Whole plant	Ethanol extract (95% v/v)	Wang et al. (2013)
6	Platanic acid	C_29_H_46_O_4_	Whole plant	Ethanol extract (95% v/v)	Wang et al. (2013)
7	Taraxasterol acetate	C_32_H_52_O_2_	Aerial parts	Ethanol extract (95% v/v)	Wang et al. (2013)
8	β-taraxasterol	C_30_H_50_O	Aerial parts	Ethanol extract (95% v/v)	Wang et al. (2013)
9	Pseudotaraxasterol	C_30_H_50_O	Whole plant	Ethanol extract (80% v/v)	Wu et al. (2012a)
10	3β, 30-dihydroxyiup-20 (29)-en-28-oic acid	C_30_H_46_O_4_	Whole plant	Ethanol extract (95% v/v)	Wang et al. (2013)
11	Taraxasteryl acetate	C_32_H_52_O_2_	Whole plant	Ethanol extract (80% v/v); supercritical fluid extraction	Wu et al. (2012a) Xiao et al. (2004)
12	Pseudotaraxasteryl acetate	C_32_H_52_O_2_	Whole plant	Ethanol extract (80% v/v)	Wu et al. (2012a)
Sesquiterpenoids					
13	Eupalinilide A	C_20_H_25_ClO_7_	Whole plant	Ethanol extract (95% v/v)	Huo et al. (2004)
14	Eupalinilide B	C_20_H_24_O_6_	Whole plant	Ethanol extract (95% v/v)	Huo et al. (2004)
15	Eupalinilide C	C_20_H_24_O_7_	Whole plant	Ethanol extract (95% v/v)	Huo et al. (2004)
16	Eupalinilide D	C_15_H_19_ClO_5_	Whole plant	Ethanol extract (95% v/v)	Huo et al. (2004)
17	Eupalinilide E	C_20_H_25_ClO_6_	Whole plant	Ethanol extract (95% v/v)	Huo et al. (2004)
18	Eupalinilide F	C_20_H_26_O_8_	Whole plant	Ethanol extract (95% v/v)	Huo et al. (2004)
19	Eupachinilide C	C_20_H_25_ClO_7_	Whole plant	Ethanol extract (95% v/v)	Huo et al. (2004)
20	Eupachifolin D	C_22_H_27_ClO_8_	Whole plant	Ethanol extract (95% v/v)	Huo et al. (2004)
21	Eupalinilide G	C_20_H_24_O_8_	Whole plant	Ethanol extract (95% v/v)	Huo et al. (2004)
22	Eupalinilide I	C_20_H_26_O_9_	Whole plant	Ethanol extract (95% v/v)	Huo et al. (2004)
23	Eupalinilide J	C_22_H_28_O_10_	Whole plant	Ethanol extract (95% v/v)	Huo et al. (2004)
24	Eupachinilide E	C_20_H_25_ClO_8_	Whole plant	Ethanol extract (95% v/v)	Huo et al. (2004)
25	Eupalinilide H	C_20_H_25_ClO_7_	Whole plant	Ethanol extract (95% v/v)	Huo et al. (2004)
26	2α-hydroxyeupatolide	C_15_H_20_O_4_	Whole plant	Ethanol extract (95% v/v)	Huo et al. (2004)
27	3-deacetyleupalinin A	C_20_H_26_O_7_	Whole plant	Ethanol extract (95% v/v)	Huo et al. (2004)
28	Heliangine	C_40_H_56_O_35_	Whole plant	Ethanol extract (95% v/v)	Huo et al. (2004)
29	8β-(4' -hydroxytigloyloxy)-3β,14-dihydroxy-6βH,7αH-germacra-1 (10)Z,4Z,11 (13)-trien-6,12-olide	C_20_H_25_O_7_	Whole plant	Ethanol extract (95% v/v)	Huo et al. (2004)
30	8β-tigloyloxy-3β,14-dihydroxy-6βH,7αH-germacra-1 (10)Z,4E,-11 (13)-trien-6,12-olide	C_20_H_25_O_6_	Whole plant	Ethanol extract (95% v/v)	Huo et al. (2004)
31	8β-tigloyloxy-2,3-seco-6βH,7αHhelianga-4Z,11 (13)-diene-3,10β; 6,12-diolid-2-oic acid	C_20_H_21_O_8_	Whole plant	Ethanol extract (95% v/v)	Huo et al. (2004)
32	Eupalinilide K	C_15_H_20_O_5_	Whole plant	Ethanol extract (95% v/v)	Huo et al. (2006)
33	Eupalinilide L	C_21_H_28_O_7_	Whole plant	Ethanol extract (95% v/v)	Huo et al. (2006)
34	3β-hydroxy-8β-(4′-hydroxytigloyloxy)-costunolide	C_20_H_26_O_6_	Whole plant	Ethanol extract (95% v/v)	Huo et al. (2006)
35	Eupalinin A	C_22_H_28_O_8_	Not stated	Not stated	Ito et al. (1979)
36	Eupalinin B	C_22_H_28_O_8_	Not stated	Not stated	Ito et al. (1979)
37	Eupalinin C	C_22_H_28_O_8_	Not stated	Not stated	Ito et al. (1979)
38	Eupalinin D	C_22_H_28_O_8_	Not stated	Not stated	Ito et al. (1979)
39	Eupalinolide A	C_24_H_30_O_9_	Aerial parts	Ethanol extract (80% v/v); Ethanol extract (95% v/v)	Yang et al. (2005b); Yang et al. (2007); Yang et al. (2009); Yan et al. (2012)
40	Eupalinolide B	C_24_H_30_O_9_	Aerial parts	Ethanol extract (80% v/v); Ethanol extract (95% v/v)	Yang et al. (2005b); Yang et al. (2007); Yang et al. (2009); Yan et al. (2012)
41	Eupalinolide C	C_22_H_28_O_8_	Aerial parts	Ethanol extract (95% v/v)	Yang et al. (2007); Yang et al. (2009)
42	Eupalinolide D	C_26_H_32_O_10_	Aerial parts	Ethanol extract (95% v/v)	Yang et al. (2007)
43	Eupalinolide E	C_24_H_28_O_9_	Aerial parts	Ethanol extract (95% v/v)	Yang et al. (2007)
44	Eupalinolide G	C_23_H_26_O_9_	Aerial parts	Ethanol extract (95% v/v)	Yang et al. (2007); Wu et al. (2012c)
45	Eupalinolide H	C_22_H_28_O_7_	Aerial parts	Ethanol extract (95% v/v)	Wu et al. (2012c); Yang et al. (2019)
46	Eupalinolide I	C_24_H_30_O_9_	Aerial parts	Ethanol extract (95% v/v)	Wu et al. (2012c); Yang et al. (2019)
47	Eupalinolide J	C_21_H_24_O_7_	Aerial parts	Ethanol extract (95% v/v)	Wu et al. (2012c); Yang et al. (2019)
48	Eupalinolide K	C_20_H_26_O_6_	Aerial parts	Ethanol extract (95% v/v)	Wu et al. (2012c); Yang et al. (2019)
49	Eupalinolide O	C_22_H_26_O_8_	Aerial parts	Ethanol extract (95% v/v)	Yang et al. (2019)
50	3β-acetoxy-8β-(4′-hydroxytigloyloxy)-14-hydroxycostunolide	C_20_H_22_O_6_	Aerial parts	Ethanol extract (95% v/v)	Yang et al. (2007); Yan et al. (2012)
Acyclic Diterpenoids					
51	3-(hydroxymethyl)-1,14,15-trihydroxy-7,11,15-trimethyl-2,6,10-hexadecatrien-13-yl acetate	C_22_H_38_O_6_	Whole plant	Ethanol extract (80% v/v)	Wu et al. (2012b)
52	3-(hydroxymethyl)-1,13,15-trihydroxy-7,11,15-trimethyl-2,6,10-hexadecatrien-14-yl acetate	C_22_H_38_O_6_	Whole plant	Ethanol extract (80% v/v)	Wu et al. (2012b)
53	3-(hydroxymethyl)-1,13,14,15-tetrahydroxy-7,11,15-trimethyl-2,6,10-hexadecatriene	C_20_H_36_O_5_	Whole plant	Ethanol extract (80% v/v)	Wu et al. (2012b)
Flavonoids					
54	Nepetin	C_16_H_12_O_7_	Whole plant	Ethanol extract (80% v/v)	Wu et al. (2012a)
55	Luteolin	C_40_H_56_O_64_	Whole plant	Ethanol extract (80% v/v)	Wu et al. (2012a)
56	Eupatrin	C_18_H_16_O_7_	Whole plant	Ethanol extract (80% v/v); supercritical fluid extraction	Xiao et al. (2004); Wu et al. (2012a)
57	Jaceosidin	C_17_H_14_O_7_	Whole plant	Ethanol extract (80% v/v); Ethanol extract (95% v/v)	Qian et al. (2004); Wu et al. (2012a)
58	Kaempferol	C_15_H_10_O_6_	Whole plant	Ethanol extract (80% v/v); supercritical fluid extraction; Ethanol extract (95% v/v)	Qian et al. (2004); Xiao et al. (2004); Wu et al. (2012a)
59	Cirsiliol	C_17_H_14_O_7_	Whole plant	Ethanol extract (80% v/v)	
60	Astragalin	C_21_H_20_O_11_	Whole plant	Ethanol extract (95% v/v)	Qian et al. (2004); Wu et al. (2012a)
61	Trifolin	C_21_H_20_O_11_	Whole plant	Ethanol extract (95% v/v)	Qian et al. (2004)
62	Hypersoide	C_21_H_20_O_12_	Whole plant	Supercritical fluid extraction; Ethanol extract (95% v/v)	Qian et al. (2004); Xiao et al. (2004);
63	Isoquercitrin	C_21_H_20_O_12_	Whole plant	Ethanol extract (80% v/v)	Wu et al. (2012a)
64	Rutin	C_27_H_30_O_16_	Aerial parts	Ethanol extract (80% v/v); supercritical fluid extraction	Xiao et al. (2004); Yang et al. 2005a; Wu et al. (2012a)
65	Linarin	C_28_H_32_O_14_	Whole plant	Ethanol extract (80% v/v)	Chu et al. (2015)
66	Quercetin	C_15_H_10_O_7_	Aerial parts	Ethanol extract (95% v/v); supercritical fluid extraction; Ethanol extract (95% v/v)	Yang et al. (2003); Qian et al. (2004); Xiao et al. (2004); Wu et al. (2012a)
67	5,8,4′-trihydroxy-7,3′-dimethoxy flavone	C_17_H_14_O_7_	Whole plant	Ethanol extract (80% v/v)	Chu et al. (2015)
Volatile oil					
68	Caryophyllene	C_14_H_22_	Whole plant	Not stated	Xiao et al. (2004)
69	Thymol	C_27_H_30_O_5_S	Whole plant	Not stated	Xiao et al. (2004)
70	Caryophyllene oxide	C_15_H_24_O	Whole plant	Not stated	Xiao et al. (2004)
71	Naphthalene,1,2,3,4,4a,5,6,8a-octahydro	C_15_H_24_	Whole plant	Not stated	Xiao et al. (2004)
72	Naphthalene,1,2,3,5,6,8a-hexahydro-4,7	C_15_H_24_	Whole plant	Not stated	Xiao et al. (2004)
73	Bicyclo [5.3.0] decane,2-methylene-5-(1-ME)	C_15_H_24_	Whole plant	Not stated	Xiao et al. (2004)
74	Amobarbital	C_14_H_24_N_2_	Whole plant	Not stated	Xiao et al. (2004)
75	α-murolene	C_15_H_24_	Whole plant	Not stated	Xiao et al. (2004)
76	(+)-Epi-Bicyclosesquiphellandrene	C_14_H_22_	Whole plant	Not stated	Xiao et al. (2004)
77	Naphthalene,1,6-dimethyl-4-(1-methyle)	C_15_H_18_	Whole plant	Not stated	Xiao et al. (2004)
78	Naphthalene,1,2,4a,5,8,8a-hexahydro-4	C_14_H_22_	Whole plant	Not stated	Xiao et al. (2004)
79	Azulene,1,4--dimethyl-7-(1-methylethyl)	C_15_H_18_	Whole plant	Not stated	Xiao et al. (2004)
80	Benzenemethanol,4-methy-l-α-phe	C_13_H_12_O	Whole plant	Not stated	Xiao et al. (2004)
81	Dodecane,2,6,11-trimethyl	C_15_H_32_	Whole plant	Not stated	Xiao et al. (2004)
82	1,6,10-Dodecatriene,7,11-Dimethyl-3-met	C_18_H_30_	Whole plant	Not stated	Xiao et al. (2004)
83	2-Pentadecanone,6,10,14-trimethyl	C_18_H_36_O	Whole plant	Not stated	Xiao et al. (2004)
84	Pentadecylic acid	C15H30O2	Whole plant	Not stated	Xiao et al. (2004)
85	Hexadecanoic acid	C16H32O2	Whole plant	Not stated	Xiao et al. (2004)
Other compounds					
86	Zhebeiresinol	C_14_H_16_O_6_	Whole plant	Aqueous extract	Zhong et al. (2017)
87	Medioresinol	C_21_H_24_O_7_	Whole plant	Aqueous extract	Zhong et al. (2017)
88	Salicifoliol	C_13_H_14_O_5_	Whole plant	Aqueous extract	Zhong et al. (2017)
89	Euparin	C_13_H_12_O_3_	Aerial parts	Supercritical fluid extraction	Xiao et al. (2004)
90	Adenosine	C_10_H_13_N_5_O_4_	Aerial parts	Ethanol extract (80% v/v)	Yang et al. (2005a)
91	Coniferyl alcohol	C_10_H_12_O_3_	Whole plant	Aqueous extract	Zhong et al. (2017)
92	Scopoletin	C_10_H_8_O_4_	Whole plant	Ethanol extract (80% v/v)	Wu et al. (2012a)
93	6,7-dimethylesculetin	C_11_H_10_O_4_	Whole plant	Ethanol extract (80% v/v)	Wu et al. (2012a)
94	5-hydroxyl-3,4-dimethy-5-pentyl-2 (5H) -furanone	C_11_H_18_O_3_	Whole plant	Aqueous extract	Zhong et al. (2017)
95	3-(2-hydroxy-4-methylbutyl)-4-methoxyaceto-phenone	C_14_H_20_O_3_	Whole plant	Aqueous extract	Zhong et al. (2017)
96	Caffeic acid	C_9_H_8_O_4_	Aerial parts	Ethanol extract (80% v/v)	Yang et al. (2005a)
97	p-hydroxy-benzaldehyde	C_7_H_6_O_2_	Whole plant	Aqueous extract	Zhong et al. (2017)
98	Daucosterol	C_35_H_60_O_6_	Aerial parts	Ethanol extract (95% v/v)	Yang et al. (2003); Chu et al. (2015)
99	β-sitosterol	C_29_H_50_O	Aerial parts	Ethanol extract (95% v/v); supercritical fluid extraction	Yang et al. (2003); Xiao et al. (2004); Chu et al. (2015)
100	Stearic acid	C_18_H_36_O_2_	Whole plant	Ethanol extract (80% v/v)	Chu et al. (2015)
101	Palmitic acid	C_16_H_32_O_2_	Aerial parts	Ethanol extract (95% v/v)	Yang et al. (2003)

### Triterpenes

The presence of triterpenes in EL has drawn the attention of researchers due to their extensive pharmacological activities in traditional medicine. (Sathya et al., 2014; Sachan et al., 2018; Rios et al., 2020). In the past few years, 12 triterpenes have been identified in EL (Table 2). Their chemical structures are shown in Figure 2. Wang *et al.* isolated seven triterpene compounds from EL for the first time: alphitolic acid, eucalyptic acid B, eucalyptolic acid, 3β,30-dihydroxyiup-20 (29)-en-28-oic acid, eucalyptic acid, platanic acid, maslinic acid, and betulinic acid (Wang et al., 2013). In addition, taraxasteryl acetate, β-taraxasterol, pseudotaraxasterol, taraxasterol acetate, and pseudotaraxasteryl acetate were identified (Yang et al., 2003; Wu et al., 2012a).

### Sesquiterpenoids

In EL, sesquiterpenoids are commonly considered to be the most important bioactive components. To date, 38 sesquiterpenoids have been identified in EL (Table 2). The structures of these sesquiterpenoids are shown in Figure 3. In 1979, Ito et al. found four new sesquiterpene lactones from EL and named them eupalinins A-D (Ito et al., 1979). Huo et al. isolated 21 sesquiterpene lactones from the plant, including eupalinilides A-J, 2α-hydroxyeupatolide, eupachinilide C, eupachifolin D, 3-deacetyleupalinin A, eupachinilide E, heliangine, eupallnilides K and L, and others (Huo et al., 2004; Huo et al., 2006). Subsequently, 3β-acetoxy-8β-(4′-hydroxytigloyloxy)-14-hydroxycostunolide, 3β-hydroxy-8β-(4′-hydroxytigloyloxy)-costunolide, eupalinolides A-E, G-K and O were identified in the plant (Yang et al., 2005b; Yang et al., 2007; Wu et al., 2012c; Yan et al., 2012; Yang et al., 2019).

### Acyclic Diterpenoids

To date, only three acyclic diterpenoids isolated from EL have been identified (Table 2): 3-(hydroxymethyl)-1,14,15-trihydroxy-7,11,15-trimethyl-2,6,10-hexadecatrien-13-yl acetate; 3-(hydroxymethyl)-1,13,15-trihydroxy-7,11,15-trimethyl-2,6,10-hexadecatrien-14-yl acetate; and 3-(hydroxymethyl)-1,13,14,15-tetrahydroxy-7,11,15-trimethyl-2,6,10-hexadecatriene (Wu et al., 2012b; Zhong et al., 2017). Figure 4 shows the chemical structures of acyclic diterpenoids in EL.

### Flavonoids

To date, 15 flavonoids have been isolated from EL (Table 2; Figure 5). Qian et al. identified 6 flavonoids in EL for the first time: jaceosidin, kaempferol, quercetin, astragalin, trifolin, and hypersoide (Qian et al., 2004). Luteolin, isoquerecitrin, rutin, cirsiliol, linarin, quercetin, 5,8,4′-trihydroxy-7,3′-dimethoxy flavone, nepetin and eupatrin were subsequently isolated from the plant (Yang et al., 2003; Xiao et al., 2004; Yang et al., 2005a; Wu et al., 2012a; Chu et al., 2015).

### Volatile Oil

Phytochemical studies on the volatile oil of EL are still preliminary. Chen et al. analyzed the constituents of volatile oil from the flowers of EL. Eighteen compounds were identified, including thymol, caryophyllene, 1,6,10-dodecatriene,7,11-dimethyl-3-met, dodecane,2,6,11-trimethyl, α-murolene, (+)-epi-bicyclosesquiphellandrene, naphthalene,1,6-dimethyl-4-(1-methyle), caryophyllene oxide, benzenemethanol,4-methy-l-α-phe, amobarbital, 2-pentadecanone,6,10,14-trimethyl, pentadecylic acid, hexadecanoic acid, and others (Table 2; Figure 6) (Chen and Yao, 2006).

### Others

In addition to the constituents mentioned above, other types of compounds have also been identified from EL, such as fatty acids, sterols, coumarins, and alkaloids (Table 2; Figure 7). The identified compounds include n-hexadecane acid (Yang et al., 2003; Wu et al., 2012a), β-sitosterol (Yang et al., 2003; Chu et al., 2015), daucosterol (Yang et al., 2003; Chu et al., 2015), caffeic acid (Yang et al., 2005), adenosine (Yang et al., 2005), vanillic acid (Chu et al., 2015), palmitic acid (Chu et al., 2015), scopoletin (Wu et al., 2012a), 6,7-dimethylesculetin (Wu et al., 2012a), and butanoic acid (Wu et al., 2012a). In addition, zhebeiresinol, medioresinol, salicifoliol, 3-(2-hydroxy-4-methylbutyl)-4-methoxyaceto-phenone, coniferyl alcohol, and p-hydroxy-benzaldehyde were also identified from EL (Zhong et al., 2017).

## Pharmacological Effects

**FIGURE 8:**
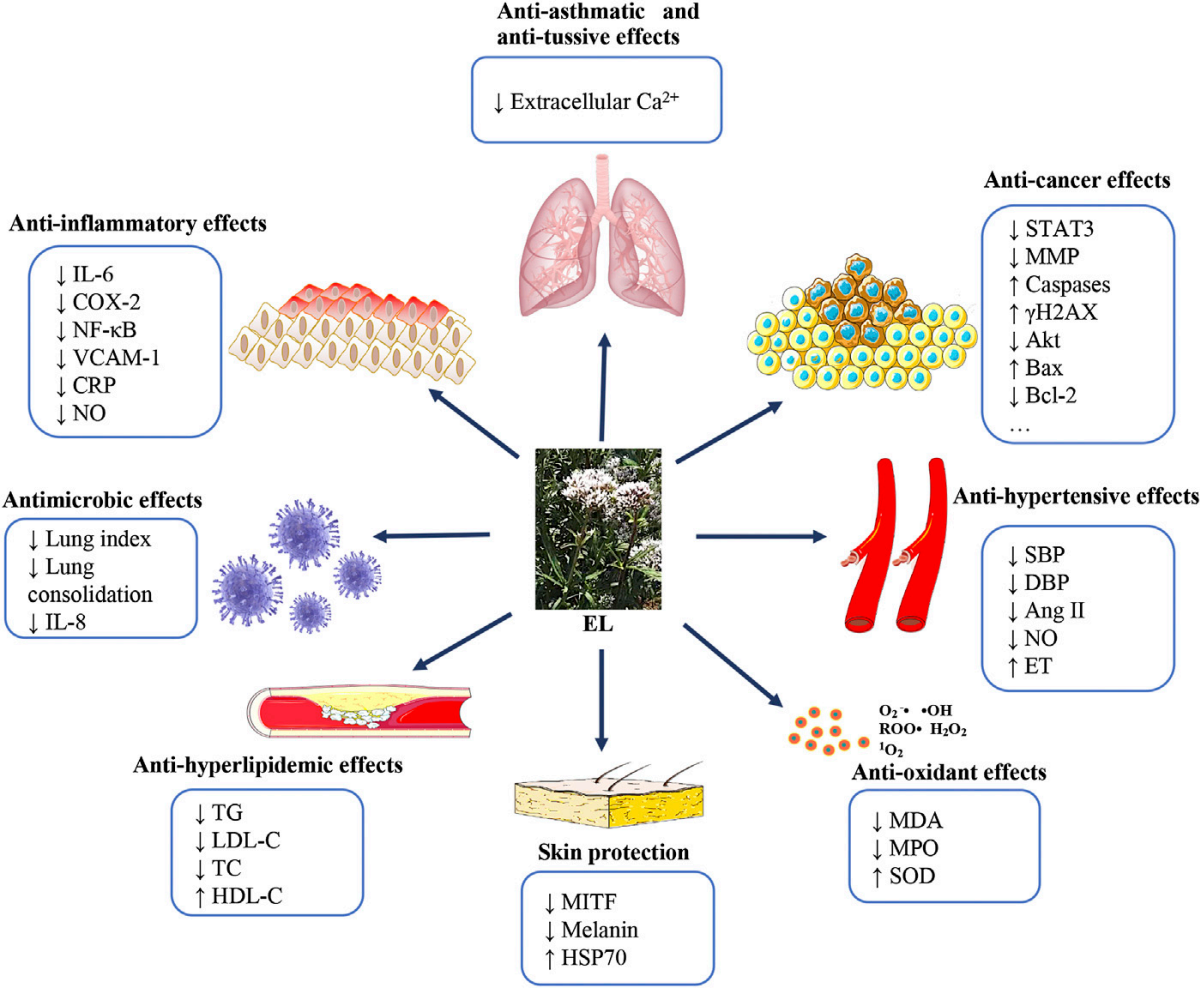
Pharmacological effects of EL according to the reported studies.

As a well-known traditional Chinese medicine, EL has been extensively applied to treat diseases of the respiratory systems. Numerous modern studies have demonstrated that EL and its constituents exhibit potent effects in ameliorating respiratory diseases (Zhu et al., 2012; Su et al., 2016; Zhang, 2017). The pharmacological effects of EL are closely connected with its anti-asthmatic, anti-tussive, and anti-inflammatory functions (Figure 8). Other diverse functions, including anti-hyperlipidemic, anti-hypertensive, anti-virus, anti-tumor, and protective effects on skin damage, were demonstrated in many studies (Figure 8). However, to date, studies of the biological activities of EL are quite preliminary and the related mechanisms of action are not well elucidated. It is unclear which constituents are responsible for the observed biological activities.

### Anti-Asthmatic and Anti-Tussive Effects

Respiratory diseases are increasing rapidly and drastically, along with environmental pollution (Verschakelen and Demedts, 1995; Mendes et al., 2020). Existing therapies for respiratory diseases are not highly effective; therefore, it is necessary to develop novel agents. The effects of EL on relieving cough and asthma have been extensively reported (Zhou et al., 2001; Tang et al., 2002; Luo et al., 2008). Pharmacodynamic studies have demonstrated the anti-tussive and anti-asthmatic activities of EL (Luo et al., 2008). It was reported that the water fraction of EL (9.334 g/kg; i.g.) could significantly reduce the cough frequency in a mouse cough model induced by concentrated ammonia. The inhibition rate was around 37.4% (*p* < 0.05). Significant anti-asthmatic activities of the water fraction (0.467 g/kg; i.g.) and petroleum ether fraction (0.329 g/kg; i.g.) were also reported in the study. The latency of asthma was significantly prolonged by treatment with the water fraction (135.41%) and petroleum ether fraction (135.63%). Experiments also documented that the fractions of petroleum ether (1.258 g/kg; i.g.), chloroform (1.421 g/kg; i.g.) and ethyl acetate (1.267 g/kg; i. g.) possessed significant expectorant activity. Compared with the control group, the phenol red secretions were increased to 146.1% (petroleum ether fraction), 165.0% (chloroform fraction), and 131.7% (ethyl acetate fraction), respectively (Luo et al., 2008). Consistent with this, Zhou et al. (2001) also identified the anti-tussive activity of the water extract of EL in ammonia- and citric acid–induced animal models. Their results demonstrated that cough latency was markedly prolonged (6.59 fold, *p* < 0.001) and cough frequency was significantly decreased (inhibition rate 80.46%, *p* < 0.001) on treatment with EL extract (45.1 g [herb]/kg). Anti-asthmatic and expectorant activities of EL (45.1 g [herb]/kg; i.g.) were also demonstrated in *in vivo* models (Zhou et al., 2001). Tang et al. (2002) investigated the possible anti-asthmatic mechanisms of EL. Studies reported that the EL extract (18.4 g [herb]/L; i.g.) exerted an inhibitory effect on the contraction of tracheal smooth muscle via inhibiting the inflow of extracellular Ca^2+^, indicating the potent anti-asthmatic mechanism of EL (Tang et al., 2002). The above studies demonstrate that EL shows potential anti-asthmatic and anti-tussive effects in *in vivo* models. The inhibition of the inflow of extracellular Ca^2+^ could be one of the main mechanisms for its anti-asthmatic activity.

### Anti-inflammatory Effects

Inflammation is a critical pathophysiological process in many chronic diseases. The anti-inflammatory activities of numerous natural medicines have been demonstrated (Li et al., 2019; Barragan-Zarate et al., 2020). Studies have documented the anti-inflammatory functions of EL in various models, such as acute lung injury (ALI) and xylene-induced mouse models. The information on anti-inflammatory studies of EL is summarized in Table 3. ALI, induced by diverse predisposing causes, is characterized by acute, progressive respiratory distress and persistent hypoxemia (Guo et al., 2020; Yang et al., 2020). To the best of our knowledge, the inflammatory response is considered as one of the most important factors in the development of ALI (Yu et al., 2015). Therefore, drugs with anti-inflammatory activity are conventionally used clinically for ALI therapy. In the traditional Chinese medicine system, studies have identified the functions of EL on prevention of ALI (Yang et al., 2010; Chu et al., 2015; Li et al., 2019; Chu et al., 2016; Yu et al., 2015; Huang et al., 2019). The mechanisms are mainly based on its anti-inflammatory and antioxidant functions. Experiments demonstrated that EL treatment (22 g/kg; i.g.) significantly reduced the expression levels of inflammatory factors and increased the level of arterial oxygen partial pressure in an ALI model. Also, oleic acid-induced elevation of lung index was significantly inhibited in EL-treated groups (22 g/kg; i.g.) (Yang et al., 2010). In an LPS-induced ALI model, fractions (of chloroform, ethyl acetate, n-butanol, water layer, flavonoids and sesquiterpenes) from EL significantly decreased the lung wet/dry (W/D) ratio and the levels of inflammatory factors, and attenuated pathological changes in the lung (Chu et al., 2015; Chu et al., 2016; Yu et al., 2015; Huang et al., 2019; Li et al., 2019). However, Li et al. (2019) demonstrated that the petroleum ether fraction of EL (30 mg/kg; i.g.) was not effective in LPS-induced ALI models. Jaceosidin, a flavonoid isolated from EL, was also found to be effective on ALI. After administration of jaceosidin (15, 30, and 60 mg/kg; i.g.), suppression of COX-2 and NF-κB was observed (Huang et al., 2019).

**TABLE 3:** Anti-inflammatory effects of EL and its potential mechanisms.

Animal	Protocol	Treatment	Major findings	Interpretation	Ref.
Wistar rats	6 h oleic (0.11 ml/kg, i.v.)	Pre-treatment with EL extracts (6, 11, and 22 g/kg, i.g.) for 2 weeks	↓ TNF-α ↓ IL-6 ↓ IL-8 ↑ PaO2	EL exhibited protective effects on ALI in rats via reducing inflammation	Jiang et al. (2007)
Wistar rats	6 h oleic (0.1 ml/kg, i.v.)	Pre-treatment with EL extracts (6, 11, and 22 g/kg, i.g.) for 2 weeks	↓ Lung index ↓ Lung W/D ratio ↓ Lung vascular permeability ↓ protein level in BALF	Pretreatment of EL showed beneficial effects on ALI induced by oleic acid in rats	Yang et al. (2010)
Kunming balb/c mice	LPS (1 mg/ml, i.v.) 6 h	EL extracts (13.5, 27, and 54 mg/kg, i.g.) for 1 week	↓ IL-6 ↓ Complement deposition ↓ TNF-α ↓ Lung W/D ratio ↓ NO ↓ proteins in BALF ↓ C3 ↓ IL-1β	EL extracts could significantly attenuate ALI via inhibiting inflammatory factors	Chu et al. (2015)
Kunming balb/c mice	LPS (1 and 2 mg/ml)	EL extracts (80 mg/kg, i.g.) for one week	↓ NO ↓ C3 ↓ TNF-α ↓ Lung W/D ratio ↓ IL-6 ↓ IL-1β	Mechanism of action of various chemical fractions of EL on ALI in mice was related to inhibiting inflammatory factors, reducing complement C3 and anti-oxidation	Li et al. (2017)
Male balb/c mice	6 h LPS (2 mg/kg, i.t.)	EUP-SQT (15, 30, and 60 mg/kg, i.g.) for 1 week	↓ NO ↓ protein in BALF ↓ C3 ↓ C3c ↓ Lung W/D ratio ↓ TNF-α ↓ IL-6 ↓ IL-1β	EUP-SQT markedly alleviated ALI through decreasing pro-inflammatory factors and complement	Yu et al. (2015)
Male Balb/c mice	6 h LPS (2 mg/kg, i.t.)	EUP-FLA (10, 20, and 40 mg/kg, i.g.) for 1 week	↓ Lung W/D ratio ↓ NO ↓ protein in BALF ↓ C3 ↓ C3c ↓ TNF-α ↓ IL-6 ↓ IL-1β	EUP-FLA alleviated ALI through decreasing pro-inflammatory factors and regulating the activity of NO, SOD, and MPO.	Chu et al. (2016)
Male Balb/c mice	24 h LPS (2 mg/kg)	Jaceosidin (15, 30, and 60 mg/kg, i.g.)	↓ Lung W/D ratio ↓ NO ↓ protein in BALF ↓ C3 ↓ C3c ↓ NF-κB ↓ COX-2 ↓ TNF-α ↓ IL-6 ↓ IL-4 ↓ IL-1β	Jaceosidin dampened the inflammation and reduced complement and antioxidant activity in ALI.	Huang et al. (2019)
Male ICR mice	Xylene (40 μL)	WE (1.8 g/kg), EEF (1.4 g/kg), SQT (0.47 g/kg) pretreated for 30 min	↓ Degree of edema formation	Sesquiterpene lactones in EL could be promising anti-inflammatory agents	Wang et al. (2017)
RAW 264.7 cells	LPS (1 μg/ml) for 24 h	Eupalinolide A-C, AHH, eupalinolide K-M, THP (2.5, 10, and 40 μM)	↓ TNF-α ↓ IL-6	Sesquiterpene lactones in EL showed significant anti-inflammatory functions	Wang et al. (2017)
RAW 264.7 cells	LPS (1 μg/ml) for 24 h	Zhebeiresinol, medioresinol and salicifoliol (40 μM)	↓ IL-6	Compounds showed anti-inflammatory activities	Zhong et al. (2017)
Male rabbits	High fat diet	EL (8.34, 4.17, and 2.08 g/kg) for 10 weeks	↑ NO ↓ CPR ↓ VCAM-1 ↑ TG	EL exerted protective and therapeutic functions by alleviating inflammatory factors	Wang et al. (2012)

Abbreviations: ALI, Acute lung injury; EEF, Ethanol eluting fraction; i.v., Intravenous; i.g., Intragastrical; i.t., Intratracheally; EL, Eupatorium lindleyanum DC.; LPS, Lipopolysaccharide; WE, Water extract; SQT, The EtOH-H2O 85:15 solution; EUP-SQT, Sesquiterpenes fraction of Eupatorium lindleyanum DC.; AHH, 3β-acetoxy-8β-(4′-hydroxytigloyloxy)-14-hydroxylcostunolide; THP, 2α-hydroxyeupatolide.

Besides the ALI models, the anti-inflammatory functions of EL have also been demonstrated in many other models. In a xylene-induced mouse model, Wang et al. investigated the anti-inflammatory functions of different fractions extracted from EL. Results demonstrated that the sesquiterpene fraction (0.47 g/kg; i.e.) reduced xylene-induced ear edema (21.53%, *p* < 0.01). *In vitro*, compounds (2.5, 10, and 40 μM) from EL, such as eupalinolide L, eupalinolide M, and 2α-hydroxyeupatolide, could significantly inhibit inflammatory factors in RAW 264.7 cells (Wang et al., 2017). Experiments also demonstrated the anti-inflammatory activities of zhebeiresinol, medioresinol, and salicifoliol from EL. At 40 μM, treatment with the compounds significantly inhibited IL-6 production. The inhibition rates were 41.6, 74.7, and 35.0%, respectively (Zhong et al., 2017). Importantly, the anti-inflammatory functions of EL were also demonstrated in a rabbit atherosclerosis model. Results indicated that the expression of C-reactive protein (CRP) and mRNA expression of VCAM-1 in EL-treated groups was markedly decreased. In addition, increased NO content was significantly observed in EL-treated groups, which might be a potential anti-inflammatory mechanism of the EL extract in atherosclerosis (Wang et al., 2012).

The above studies demonstrated that the fractions and constituents from EL showed significant anti-inflammatory activities both *in vitro* and in *in vivo* model systems. The potential mechanisms were mainly related to the suppression of inflammatory factors, including IL-6, COX-2, and NF-κB.

### Antioxidant Effects

An imbalance between the production of oxidants and antioxidants often results in oxidative stress (Kemp et al., 2008). Overproduction of oxidants such as malonic aldehyde (MDA) and depletion of SOD and GSH resulted in inflammatory processes and oxidative damage (; Sun et al., 2016; Mo et al., 2020). The antioxidant functions of EL were widely shown in different ALI models (Table 4). In LPS- and oleic acid–induced acute lung injury models, increased levels of SOD and reduced levels of MDA and MPO were significantly detected on treatment with EL compounds and fractions (Zhou et al., 2017). Yan et al. (2011) evaluated the antioxidant effects of various extracts from EL. Their results verified that the water extract (400 μg/ml) and residue water extract (400 μg/ml) showed significant superoxide anion radical scavenging activity (the inhibition rates were 94.55 and 95.77%) (Yan et al., 2011). The antioxidant activity of flavonoids in EL were also assessed with the DPPH (2,2-diphenyl-1-picry-hydrazyl radical) method. Flavonoids showed powerful scavenging activities on DPPH free radical with an IC_50_ of 10.92 mg/ml (Wang et al., 2010). In addition, Yan et al. developed an ultrasonic-microwave synergistic extraction method to increase the yield rate of antioxidants from EL, which was found to be more effective than other methods (Yan et al., 2013). The above data indicated that EL exhibited significant antioxidant effects in various models through increasing the level of SOD and reducing the levels of MDA and MPO.

**TABLE 4:** Antioxidant activities of EL and its potential mechanisms.

Animal	Protocol	Treatment	Major findings	Interpretation	Ref.
Wistar rats	6 h oleic (0.11 ml/kg, i.v.)	Pre-treatment with EL extracts (6, 11, and 22 g/kg, i.g.) for 14 days	↓ MDA ↑ SOD	EL exhibited protective effects on ALI in rat via reducing oxidative stress	Jiang et al. (2007)
Kunming Balb/c mice	LPS (1 mg/ml, i.v.) for 6 h	EL extracts (13.5, 27, and 54 mg/kg, i.g.) for one week	↓ MPO ↑ SOD	EL extracts could significantly attenuate ALI through decreasing inflammatory factors and complement	Chu et al. (2015)
Kunming Balb/c mice	LPS (1 and 2 mg/ml)	EL extracts (80 mg/kg, i.g.) for one week	↓ MPO ↑ SOD	Mechanism of action of various chemical fractions of EL on ALI in mice is related to inhibiting inflammatory factors, reducing complement C3 and anti-oxidation	Li et al. (2017)
Male Balb/c mice	6 h LPS (2 mg/kg, i.t.)	EUP-SQT (15, 30, and 60 mg/kg, i.g.) for one week	↓ MPO ↑ SOD	The antioxidant activity of EUP-SQT played an important role in LPS-induced ALI.	Yu et al. (2015)
Male Balb/c mice	6 h LPS (2 mg/kg, i.t.)	EUP-FLA (10, 20, and 40 mg/kg, i.g.) for one week	↓ MPO ↑ SOD	The antioxidant activity of EUP-FLA played a key role in LPS-induced ALI.	Chu et al. (2016)
Male Balb/c mice	24 h LPS (2 mg/kg)	Jaceosidin (15, 30, and 60 mg/kg, i.g.)	↓ MPO ↑ SOD	Jaceocidin exhibited antioxidant activity during ALI.	Huang et al. (2019)
—	DPPH method; riboflavin-light-NBT system	WE, EE, RWE, PF, EF, BF and WF (25–400 μg/ml)	↑ DPPH RSA ↑ superoxide anion RSA	The extracts from EL were demonstrated to be effective antioxidant constituents	Yan et al. (2011)
—	DPPH method	Flavonoids	↑ DPPH RSA	The IC_50_ value of the flavonoids was 10.922 μg/ml	Wang et al. (2010)

Abbreviations: WE, Water extract; i.v., Intravenous; i.g., Intragastrical; i.t., intratracheally; EL, Eupatorium lindleyanum DC.; PF, Petroleum ether fraction; MDA, Malonic aldehyde; SOD, Superoxide dismutase; WF, Water fraction; ALI, Acute lung injury; EUP-SQT, Sesquiterpenes fraction of Eupatorium lindleyanum DC.; RSA, Radical scavenging activity; EE, Ethanol extract; RWE, Residue water extract; LPS, Lipopolysaccharide; EF, Ethyl acetate fraction; BF, n-BuOH fraction; DPPH, 2, 2-diphenyl-1-picry-hydrazylradical

### Anti-Hyperlipidemic Effects

Hyperlipidemia, a metabolic disturbance of lipid, is one of the most lethal factors for cardiovascular and cerebrovascular diseases (Pappa et al., 2019; Zeng et al., 2019). It is conventionally characterized by abnormal elevations of LDL-C, TC, and LDL (Zhou et al., 2015). Experiments have demonstrated the anti-hyperlipidemic effects of extracts and compounds from EL. Kaempferol (100 and 300 mg/kg; i.g.) and total flavonoids (50 and 100 mg/kg; i.g.) from EL could markedly lower serum blood lipids, hemorheological parameters, and blood viscosity (He et al., 2014). Most importantly, the contents of TG, LDL-C, and TC were markedly reduced while HDL-C was significantly raised by treatment with EL. These results suggest that EL shows marked preventive and therapeutic effects on hyperlipidemia in rats (Zhou et al., 2017). Consistent with this, Wang et al. (2009) also demonstrated the anti-hyperlipidemia effects of EL (11.28, 22.56, and 45.12 g/kg; i.g.) in an experimental hyperlipemia model. In addition, studies demonstrated that EL (25 g/kg; i.g.) exerted hypolipidemic activity via regulating low density lipoprotein receptor (LDLR) mRNA in hyperlipidemic rats (Chen et al., 2009). The above experiments demonstrate that EL exerts anti-hyperlipidemic effects through regulating LDLR, increasing the content of HDL-C, and decreasing the levels of TG, LDL-C, and TC.

### Anti-Hypertensive Effects

The anti-hypertensive effects of EL were demonstrated in different animal models. Studies demonstrated that the water extract of EL (5 and 10 mg [herb]/mL; i.g.) could significantly suppress the vasoconstriction of the vascular vessels and regulate extracellular calcium influx and intracellular calcium release (Jiang et al., 2007). Moreover, the anti-hypertensive effects of the water decoction of EL were demonstrated in a spontaneously hypertensive rat (SHR) model. SHRs were treated with a water decoction of EL (16.36, 32.73, and 65.45 g/kg; i.g.) for 7 weeks. Rats in the EL-treated groups showed markedly lower systolic blood pressure (SBP) and diastolic blood pressure (DBP) of the caudal artery, indicating the anti-hypertensive effects of the water decoction of EL. Also, decreased levels of NO and angiotensin (Ang Ⅱ) and increased level of endothelin (ET) in serum were observed, suggesting the possible anti-hypertensive mechanisms of EL (Chi, 2016). These experiments demonstrated that EL exerted significant anti-hypertensive activities *in vivo*. The suppression of Ang Ⅱ and upregulation of ET could be the potential mechanisms of action.

### Antimicrobial Effects

The antimicrobial effects of the EL preparations were reported in many studies (Peng et al., 2008a; Dou et al., 2011). Studies were mainly focused on the compound Yemazhui capsule, in which EL was one of the most important herbs. Peng et al. (2008a) confirmed that the compound Yemazhui capsule (0.1, 0.2, and 0.4 g/ml; i.g.) could effectively inhibit the influenza virus, prolong the lifespan (34.0%), decrease the lung index (16.7%), and inhibit lung consolidation *in vivo* (Peng et al., 2008a). Furthermore, they also demonstrated the anti-virus effects of compound Yemazhui capsule (0.1, 0.2, and 0.4 g/ml; i.g.) in parainfluenza virus (Peng et al., 2008a). Dou et al. (2011) also demonstrated the anti-virus effects of compound Yemazhui capsule on respiratory syncytial virus (RSV). Results showed that the compound Yemazhui capsule (31.3 mg/L) significantly inhibited the RSV infection. The inhibition rate was around 60.81%. Interestingly, marked suppression of IL-8 in RSV-infected A549 cells was observed. Peng et al. (2008b) also evaluated the anti-bacterial activities of the compound Yemazhui capsule *in vivo*. Results showed that the compound Yemazhui capsule (8 g [herb]/kg) significantly reduced the mortality of mice after infection with streptococci (Peng et al., 2008b). The mortality was markedly decreased from 70.00% (the control group) to 31.58% (the compound Yemazhui capsule group). These studies demonstrated that the compound Yemazhui capsule showed significant antimicrobial effects in *in vitro* and *in vivo* model systems. However, the exact antimicrobial mechanisms of action are still unclear.

### Anti-Tumor Activities

Recently, the anti-tumor activities of EL have been widely investigated. Studies have demonstrated that the compounds and fractions from EL showed significant anti-tumor activities (Table 5). The related mechanisms are quite complicated. F1012-2 (5, 10, and 20 μg/ml), an active extract of EL, could significantly inhibit the growth of TNBC cells. Studies demonstrated that F1012-2 suppressed cancer cells through induction of apoptosis and cell cycle arrest (G2/M) (Tian et al., 2018). Significant inhibition of Akt and activation of p38 signaling pathways were observed in the study. Yang et al. (2007) found that the compounds from EL, including eupalinolides A-E and 3β-acetoxy-8β-(40-hydroxytigloyloxy)-14-hydroxycostunolide, could suppress the proliferation of cancer cells. Eupalinolide O (EO), one of the main compounds in EL, was demonstrated to be effective in suppressing the growth of MDA-MB-468 cells. The inhibitory effects of EO (5, 10, and 20 μM) were related to apoptosis induction (Yang et al., 2016). Moreover, some studies also demonstrated that Eupalinolide J (EJ) exhibited significant anticancer activity in TNBC cells. Experiments documented that EJ suppressed the proliferation of cancer cells through induction of apoptosis, disruption of MMP, and suppression of the STAT3 pathway (Lou et al., 2019; Yang et al., 2019). Wu et al. (2020) also demonstrated the anticancer effects of EJ (2.5, 5, 10, and 20 μM) in prostate cancer cells. Their results indicated that EJ suppressed the growth of prostate cancer cells via induction of DNA damage responses. The expression levels of p-Chk1, p-Chk2, and γH2AX were remarkably increased with the treatment of EJ (Wu et al., 2020). The above results indicated that the fractions and compounds isolated from EL showed significant anticancer activities. The possible mechanisms were largely associated with induction of apoptosis, cell cycle arrest, disruption of MMP, induction of DNA damage responses, and suppression of STAT3 and Akt signaling pathways.

**TABLE 5:** Anti-tumor activities of EL and its potential mechanisms.

Models	Treatment	Major findings	Interpretation	Ref.
MDA-MB-468 cells	EO (0, 1, 5, 10, and 20 μM) for 24, 48, and 72 h	↓ cell viability ↑ cells in G2/M phase ↑ apoptosis ↓ MMP ↑ Caspase-3/-8/-9 ↓ Akt ↑ bax ↓ Bcl-2 ↑ bad ↓ bcl-xl	EO inhibited cancer cells by caspase-dependent apoptosis induction	Yang et al. (2016)
PC-3 and DU-145 cells	EJ (0, 2.5, 5, 10, and 20 μM) for 24, 48, and 72 h	↓ cell viability ↑ cells G2/M phase ↑ apoptosis ↓ MMP ↑ Caspase-3/-9 ↑ *p*-Chk1 ↑ *p*-Chk2 ↑ γH2AX	EJ suppressed prostate cancer cells via DNA damage induction	Wu et al. (2020)
MDA-MB-231 and MDA-MB-468 cells	EJ (0, 5, 10, 20, and 30 μM) for 24, 48, and 72 h	↓ cell viability ↑ cells in G2/M phase ↑ apoptosis ↓ MMP ↑ Caspase-3/-8/-9 ↓ STAT3	EJ inhibited the proliferation of cancer cells through blocking STAT3 pathway	Lou et al. (2019)
MDA-MB-231 cells	F1012–2 (0, 1, 5, 10, and 20 μg/ml) for 24, 48, and 72 h	↓ cell viability ↑ cells in G2/M phase ↑ apoptosis ↓ MMP ↑ Caspase-3/-8/-9 ↓ Akt ↑ p38 ↑ autophagy	F1012–2 suppressed cell proliferation through affecting multiple signaling pathways	Tian et al. (2018)
MDA-MB-231 and MDA-MB-468 cells	EJ, EO, EI, EK, EH, and EG (10 μM) for 24 h	↓ cell viability ↓ STAT3	EJ had a notable inhibitory effect on STAT3 activation in TNBC cells	Yang et al. (2019)

Abbreviations: EO, Eupalinolide O; MMP, Mitochondrial membrane potential; EJ, Eupalinolide J; EI, Eupalinolide I; EK, Eupalinolide K; EH, Eupalinolide H; EG, Eupalinolide G; TNBC, Triple‐negative breast cancer.

### Protective Effects on Skin Damage

Protective effects of EL on skin damage have been demonstrated. According to the reports, microphthalmia-associated transcription factor (MITF) and heat shock protein 70 (HSP70) play a key role in the production of melanin (Park et al., 2018; Stangl et al., 2018). Experiments demonstrate that EL extract (0.08 mg/ml) could significantly suppress MITF, melanin production, and tyrosinase activity in HSP70-overexpressed cells (Yamashita et al., 2010). Their further study documented that eupalinolide A (EA) and eupalinolide B (EB) were the active HSP-inducers in the EL extracts. After treatment with EA (10 μg/ml) and EB (10 μg/ml), the level of HSP70 in the skin was significantly up-regulated. Importantly, the damage induced by UVB radiation was significantly alleviated, suggesting the effects of both compounds on preventing skin damage and melanin production (Yamashita et al., 2012).

## Toxicology

To date, information on toxicological studies of EL is still limited. Li et al. examined the effects of EL in the nervous, cardiovascular, and respiratory systems of rats. Results demonstrated that the extracts of EL did not show any significant effects in rats even at a concentration of 45 g (herb)/kg (Li et al., 2005). Zhou et al. investigated the general pharmacological actions and toxicity of EL. The autonomous movement, cardiovascular and respiratory reactions, and acute and chronic toxicity were tested in rats and mice. Interestingly, EL did not show any significant effects in these tests. The LD_50_ value was 225.6 g (herb)/kg. No toxic reaction was found in a chronic toxicity test. It was concluded that EL had no obvious influence on normal physiological action and tissues and organs in animals (Zhou et al., 2005). Importantly, clinical findings also supported the above results (Su et al., 2016). The above studies suggested that EL was safe in traditional application. However, further toxicological studies are still needed to test its clinical safety.

## Pharmacokinetics

To date, there are few reports on the pharmacokinetics of EL. Pharmacokinetic studies should be prioritized to better understand the absorption, distribution, metabolism, and excretion of the active constituents in EL. UHPLC-TOF-MS was used to analyze the mass spectrometric decomposition and metabolic transformation of eupalinolide F in rats. It was demonstrated that eupalinolide F forms a series of 55 related metabolites via undergoing multiple biotransformation pathways (Qin et al., 2019). In addition, Zhang et al. found that Maximum concentrations (C_max_) of HYP, EA, and EB in plasma occurred at 1.80 ± 0.84, 1.00 ± 0.62, and 0.57 ± 0.09 h after treatment with EL extract (625 mg/kg) in rats. C_max_ values of EA, EB, and HYP were 144.73 ± 35.90, 371.25 ± 63.91, and 689.60 ± 80.88 ng/ml, respectively. Studies demonstrated that C_max_, AUC_0−t_, and AUC_0−∞_ rose in a dose-dependent way with increased dosages of EL fractions (Zhang et al., 2015).

## Conclusion and Future Prospects

In conclusion, this review emphasizes the significance of the herb in traditional Chinese medicine and summarizes the findings of published studies. The traditional uses, botany, phytochemistry, pharmacological activities, toxicity, and pharmacokinetics of EL have been stated in this paper. EL has been extensively used clinically to treat respiratory diseases. It exhibits anti-inflammatory, antioxidant, anti-tumor, antimicrobial, anti-hyperlipidemic, and anti-hypertensive activities. Terpenes, especially sesquiterpenoids, are considered to be the most important constituents in EL. Moreover, the preparations of EL, including Eupatorium Kechuan powder, modified Yemazhui capsule, Jiawei Yemazhui capsule, sustained-release preparation of Yemazhui, and Yemazhui syrup, have been widely applied in traditional prescriptions to cure many kinds of diseases. Hence, EL has an important role in the traditional Chinese medicine system.

To date, considerable research on EL has been done in various fields, especially in phytochemistry and pharmacological activities. However, challenges still exist. First, although a large number of compounds have been identified in the plant, studies are still necessary to explore all new compounds in EL. Second, various biological effects, such as anti-asthmatic, anti-tussive, anti-inflammatory, anti-tumor, anti-virus, anti-hyperlipidemic, and anti-hypertensive functions, have been widely reported. However, the exact active constituents and potential mechanisms are still unclear. Clinical studies are still necessary to further evaluate these biological functions. Third, toxicity studies, including sub-chronic or acute toxicity, are also very important to determine the safety of EL. Finally, further studies are still needed to investigate in detail the pharmacokinetics and therapeutic doses in humans to better understand the pharmacological activities of EL. This review provides useful background for researchers on the current status of EL investigation and suggests possible directions for future study.

## Author Contributions

XW, SM, FL, and YW performed the search, screened the papers, edited the tables, and wrote the text. CL designed the study, wrote, and revised the paper.

## Funding

This work was financially supported by the Zhejiang Chinese Medical University Research Fund Project (2019ZG32).

## Conflict of Interest

The authors declare that the research was conducted in the absence of any commercial or financial relationships that could be construed as a potential conflict of interest.

## Glossary

EL: *Eupatorium lindleyanum* DC.
PF: Petroleum ether fraction
CRP: C-reactive protein
WE: Water extract
ALI: Acute lung injury
EE: Ethanol extract
TC: Total cholesterol
RWE: Residue water extract
HL: Hepatic lipase
EF: Ethyl acetate fraction
BF: n-BuOH fraction
TG: Triglyceride
BALF: Bronchoalveolar lavage fluid
DPPH: 2, 2-diphenyl-1-picry-hydrazylradical
MDA: Malonic aldehyde
SOD: Superoxide dismutase
WF: Water fraction
HDL-C: High density lipoprotein cholesterol
ET: Endothelin
RSV: Respiratory syncytial virus
LDL-C: Low density lipoprotein cholesterol
SHR: Spontaneously hypertensive rat
TNBC: Triple-negative breast cancer
EO: Eupalinolide O
DBP: Diastolic blood pressure
MMP: Mitochondrial membrane potential
UV: Ultraviolet
LDLR: Low density lipoprotein receptor
SBP: Systolic blood pressure
DBP: Diastolic blood pressure
MITF: Microphthalmia-associated transcription factor
CRR: clinical response rate
EA: Eupalinolide A
EB: Eupalinolide B
UHPLC-TOF-MS: Ultra-high performance liquid chromatography-four-stage rod-time-of-flight mass spectrometry
Cmax: The maximum concentrations
HYP: Hyperoside
Ang Ⅱ: Angiotensin
SBP: Systolic blood pressure
LPS: Lipopolysaccharide
EEF: Ethanol eluting fraction
MPO: Myeloperoxidase
LPL: Lipoprotein lipase
LCAT: Lecithin cholesterol acyltransferase
